# Description of a new species, *Pintomyia dissimilis *nov. sp., a phlebotomine fossil from Dominican Republic amber (Diptera: Psychodidae: Phlebotominae)

**DOI:** 10.1186/1756-3305-2-25

**Published:** 2009-05-14

**Authors:** José Dilermando Andrade Filho, Paula Cavalcante Lamy Serra e Meira, Cristiani de Castilho Sanguinette, Reginaldo Peçanha Brazil

**Affiliations:** 1 Instituto René Rachou-Fiocruz, Av. Augusto de Lima 1715, CEP 30190-002 Belo Horizonte, MG, Brazil; 2 Departamento de Bioquímica e Biologia Molecular, Instituto Oswaldo Cruz-Fiocruz, Rio de Janeiro, RJ, Brazil

## Abstract

**Background:**

Phlebotomine sandflies are the vectors of etiological agents of leishmaniases in several areas of the world. In the Neotropical Region, the biodiversity of these insects is more than other regions, probably due the long evolutionary period of this group. Miocene amber from Dominican Republic, currently, has a record of 14 extinct species of Phlebotomine sandflies.

**Results:**

This paper describes a new fossil species of phlebotomine sandfly from amber found in Dominican Republic. This new species is based on morphological characters of a male such as 5° palpomere longer than 3° + 4°, three well-developed spines in the gonostyle, lateral lobe longer than gonocoxite and permit inclusion of the new species in the genus *Pintomyia*, series *serrana*. The paramere, with a curvature in the ventral margin, of the middle of the structure, separates the new species from the others fossils or extant species.

**Conclusion:**

The new species described in the present study named *Pintomyia dissimilis *nov. sp. is well differenciated from all known species in this genus.

## Background

Several tropical diseases are transmitted by insects and among them is included the leishmaniases, a group of diseases which the aethiological agents are species of parasite of the genus *Leishmania*. Phlebotominae sandflies are responsible for the transmission among vertebrate hosts. Transmission occurs during blood feeding of females and, up to now, around 30 species are known to be involved in the transmission of the disease [1].

This host/parasite is very old being recorded in the Cretaceous and Miocene periods, where extinct sandflies in amber have been found to be associated with protozoa described as belonging to the genus *Paleoleishmania *[2,3]. This last record is related to amber of the Dominican Republic in the Hispaniola Island, Caribbean region of Central America.

To date, in the New World, fifteen species of fossil sandflies had been formally described and most are from the Dominican Republic and only one from Mexico. All the species are within the genera *Pintomyia *Costa Lima, 1932; *Micropygomyia *Barretto, 1962; and *Psathyromyia *Barretto, 1962 [4,5].

The objective of this study was to carry out a description of one species of phlebotomine sandflies from the Dominican Republic, based on a holotype male, enclosed in amber.

## Methods

The description of the fossil species was based on direct observation under the optical microscope of the holotype specimen. The piece of amber measuring 0.9 by 0.8 by 0.5 mm, containing the male holotype and one flea. The insects were examined under the microscope and measured using an ocular micrometer calibrated for this purpose. Drawings were made using a microscope with the aid of a camera lucida and photographs taken with Konica digital equipment with a definition of 8.0 megapixels, the photographs being taken directly through the eyepiece of the microscope. The measurements are given in micrometers. Some morphological details that help to distinguish fossil from extant species have been provided. The classification system of Galati [6] was used.

## Results

### Description of Pintomyia dissimilis nov. sp. (Figs 1, 2 and 3)

**Figure 1:**
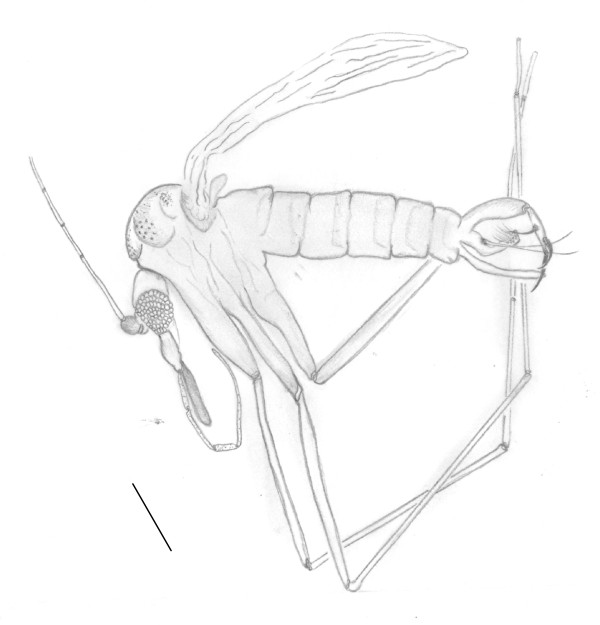
**drawing of *Pintomyia dissimilis *nov. sp. Bar = 250 μm**.

**Figure 2:**
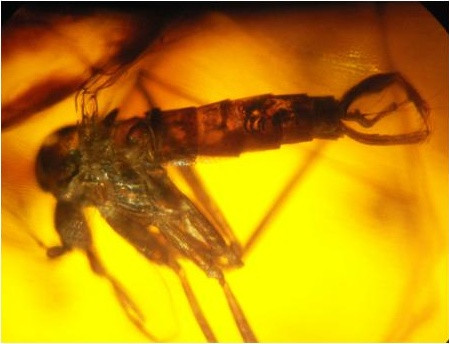
***Pintomyia dissimilis *nov. sp. – general aspect**.

**Figure 3:**
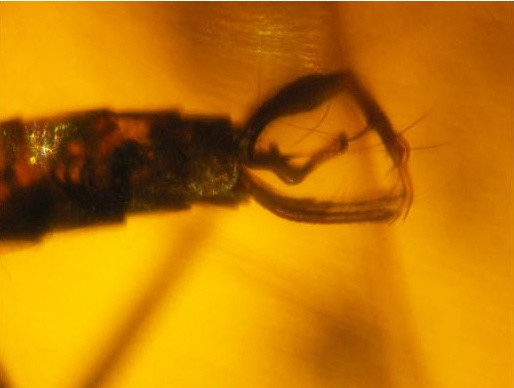
***Pintomyia dissimilis *nov. sp. – terminalia showing the different paramere**.

#### Holotype male

Total length 2.029. The coloration of the insect was indistinguishable.

#### Head (lateral view)

Clypeus 124 μm and labrum-epipharynx 235 μm long. Flagellomeres measurements: AIII 262; AIV 124; AV 110, AXVI and AXV lost in holotype. Interocular distance was not measured due to the lateral position of the head. Palpal formula 1.4.2.3.5; palpomere lengths as follows: 1° – 28, 2° – 138, 3° – 152, 4° – 83, 5° – 359. Ascoids, papillae and Newstead's spine not visible.

#### Cervix

Ventro-cervical sensillae could not be observed.

#### Thorax

Hind femur without row spine. Length of femora, tibia, basitarsi and tarsi II+III+IV+V: on foreleg 718, 800, 593 and, 718 μm; midleg 745, 952, and 662 μm (tarsi II+III+IV+V lost); hindleg 772, 1.132, 745 and, 718 μm. Due the wing position, was impossible to measure the veins sections, width and length, but Sc is free.

#### Abdomen

Gonocoxite 248 μm and 55 width, without tuft of bristles. Gonostyle 207 μm, presenting three developed spines, of which one apical, one internal on the basal third and the other external implanted close to the apical. Pre-apical bristle absent. Paramere 221 μm long, presenting strong curvature in elbow-shaped in the median region of the ventral margin and a group of small bristles at its apex. Lateral lobe long, measuring 290 length by 28 width. Lateral lobe/gonocoxite 1.17: 1. Is not visible the ejaculatory pump and genital filament throughout its length. Part of this is out of abdomen, leaving the aedeagus, the exposed area measured 304 μm, and the tip is simple.

#### Derivatio nominis

From the Latim *dissimilis *(= different) in reference to the different paramere of this species.

#### Type Material

One male holotype specimens in Miocene amber from Dominican Republic, north of Santiago, deposited in the Collection of phlebotomine of the Instituto René Rachou (Fundação Oswaldo Cruz), Belo Horizonte, MG, Brazil. In accordance with section 8.6 of the ICZN's International Code of Zoological Nomenclature, we have deposited copies of this article at the following five publicly accessible libraries: Natural History Museum, London, UK; American Museum of Natural History, New York, USA; Museum National d'Histoire Naturelle, Paris, France; Russian Academy of Sciences, Moscow, Russia; Academia Sinica, Taipei.

## Discussion

Although the ascoids have not been displayed, as well as the papillae and Newstead spines, the character set, such as palpomere 5 longer than 3 + 4; gonostyle with three spines, paramere simple and lateral lobe longer than the gonocoxite permit the specimen to be to included a new species in the genus *Pintomyia*. The absent of row spine in the hindfemur exclude the new species of the *Pintomyia *sensu stricto. *Pintomyia dissimilis *nov. sp. is close to *Pifanomyia *subgenus, series *serrana*, in which some species present three spines on the gonostyle in the same arrangement as this new fossil species.

Four extant species in series *serrana *have three well developed spines in the gonostyle, i.e. *Pintomyia orestes *(Fairchild & Trapido, 1950), *Pintomyia christophei *(Fairchild & Trapido, 1950), *Pintomyia diazi *(Gonzalves & Garcia, 1981), and *Pintomyia novoae *(Gonzalves & Garcia, 1981). All these species present tufts of bristles in the gonocoxite, absent in the fossil species.

Of 14 male fossil species described, 10 belong to the genus *Pintomyia *[5]. *Pintomyia paleopestis *(Peñalver & Grimaldi, 2005) and *Pintomyia brazilorum *Andrade Filho, Galati & Falcão, 2006 present only two developed spines in the gonostyle [7,8] while in *Pintomyia falcaorum *Brazil & Andrade Filho, 2002, *Pintomyia killickorum *(Andrade Filho, Falcão, and Brazil, 2004) *Pintomyia filipalpis *(Peñalver & Grimaldi, 2005), *Pintomyia paleotownsedi *Andrade Filho, Falcão, Galati, and Brazil, 2006, *Pintomyia paleotrichia *Andrade Filho, Brazil, Falcão, and Galati, 2007, and *Pintomyia dominicana *Andrade Filho, Galati & Brazil, 2009 this structure has four spines [5,7,9-12]

Only two phlebotomine fossil species have three developed spines in the gonostyle, i. e., *Pintomyia succini *(Peñalver & Grimaldi, 2005) and *Pintomyia miocena *(Peñalver & Grimaldi, 2005) [7]. *Pintomyia dissimilis *nov. sp. differ from both by the aspect of the paramere, that presents a strong curvature, absent in the other species.

One species, *Lutzomyia adketis *Poinar Jr 2008 was described based in one female, with the following characteristics: sc forked with the branches meeting the costa and radius veins and the shape and size of the spatulate rods on the ninth sternite [13]. Although the principals vein sections are not described, the sc is free in *Pintomyia dissimilis *and can be used to separate both species.

## Conclusion

The new species described in the present study named *Pintomyia dissimilis *nov. sp. is well differenciated from all known species in this genus. With the description of the new species, the fauna of phlebotomine sandflies fossils contains 16 species, 11 belong to the genus *Pintomyia*.

## Competing interests

The authors declare that they have no competing interests.

## Authors' contributions

JDAF, PCLSM, CCS and RPB participated in morphological analysis of the specimens. CCS and PCLSM did drawing and measurements of the new species. JDAF and RPB drafted the manuscript. All authors read and agreed with this manuscript.
